# Supplementary material for: Patterns of Intron Gain and Loss in Fungi
Source: PLoS Biol. 2004 Nov 30;2(12):e422. doi: 10.1371/journal.pbio.0020422 (PMC532390; doi:10.1371/journal.pbio.0020422)
Supplement: Table S1 — Also available at http://genes.mit.edu/NielsenEtAl/. (4.3 MB ZIP). [file pbio.0020422.st001.zip › NielsenEtAl/html/584.html]

AN4284.1.NCU01766.1.MG03153.1.FG05204.1


```
 CLUSTAL W (1.82) Multiple Sequence Alignments - Introns Inserted


Sequence 1: MG03153.1	256 aa
Sequence 2: FG05204.1	233 aa
Sequence 3: NCU01766.1	252 aa
Sequence 4: AN4284.1	188 aa
Alignment Length: 276 aa
Number Identitical Residues: 25 aa
Alignment Score (without introns) 1903


MG03153.1 	-MENHSQQP--IAGQQ--PHQSEDERLFSEFDSYPWQEDSVFL0TGLIALLGGVDKLRPD
NCU01766.1	-MEIPTQQP--STGGN-------DVAAFKKFDAYPWIKDRSFL0QGLVAMLGPLSNGFER
FG05204.1 	MSQQQSSQS--LAGADRIRALKDDDAIFTAFDTYPWTKDKNFM0SGLCAILGEPGQQDPQ
AN4284.1  	-METSSPHPRESITTAQTESRNPQADIFEQLSTYPFSTDREFA~GGLAIILGHPETPASD
          	  :  : :. .          . :   *  :.:**:  *  *   **  :**        

MG03153.1 	---PSHTDLAVHARIFYYQQ~A--RNARVDFSAYKQWAAASTARNSTDTEVLPALYWARE
NCU01766.1	---QKALGISLQARVWWYKS~R--FSIDIDRSAYENYLLPSSSPSTPSHSQIPNSPVDAQ
FG05204.1 	---ASLSDMAIHARIFYYAQ~R--IGVDIDFGSYKSWLASKTNHQPPD--VLPEEYRQRA
AN4284.1  	EEIARDDDLVLQAKCYYFIR2KNNLALPLDVATYKSWLEARRTS----------------
          	..     .: ::*: :::    ..    :* .:*:.:  .                    

MG03153.1 	QISALDGIPPANAVDAVAAVAQQYVPPPPDPAWQQAAPAQPLRVSH---ASVGGDGGTGA
NCU01766.1	LVDKLAEIQQLMGTTPVSASD------DDLPAWQVQAPKVDLLKKADDGDADGAERGANG
FG05204.1 	EANATS---------------------TAALDWQKAAPKTDLYIDR---KAAAAQSGSED
AN4284.1  	---------------------------STSIDGSRETGSSQISASSLSPTTVPASKPTSQ
          	                                 .  :    :  .  .  :  ..  :  

MG03153.1 	AP-YPAHFAELIAAVQNNQPVPGVREIPDTVIRDS0NVKPVGKMTAPKKPWELGRTSSTE
NCU01766.1	NAPYPERFNAIIELITNGTPVPGIKEIPNTVVRQP0GISPIGKMQAPQKPWEKKQQQAAA
FG05204.1 	QPNYPMGFAEMIKLIQEGKPVPGIRQIPNTIVRDP0AVKPVGARTAPRKPWEKDVSPDSS
AN4284.1  	EPTYPTSFAHIVELITTGQPVPGIQHIPDILLTG-~-QGAASAKPKRLKPWEKGETA---
          	 . **  *  ::  :  . ****::.**: ::       . .      ****        

MG03153.1 	AQGGQALQHDATVDQDFPPIDGQAQTQTSGGQP------
NCU01766.1	AN---LVTSEVMIDREFPPLPPEDEDQEHQRDQPQKMGA
FG05204.1 	T----LADLPKALDTEFPPLHDDPVASSAAGTS------
AN4284.1  	---------------------------------------
          	
```
